# Prediction of conelet mortality rates in interior lodgepole pine using phenomic, nutritional, and environmental data

**DOI:** 10.3389/fpls.2026.1845181

**Published:** 2026-10-08

**Authors:** Julian Garcia-Abadillo, Julio Isidro y Sánchez, Diego Jarquin, Esteban Galeano, Barb R. Thomas

**Affiliations:** 1 Agronomy Department, University of Florida, Gainesville, FL, United States; 2 Centro de Biotecnología y Genómica de Plantas, Universidad Politécnica de Madrid, Pozuelo de Alarcón, Spain; 3 Department of Forestry, Mississippi State University, Mississippi, MS, United States; 4 Department of Renewable Resources, University of Alberta, Edmonton, AB, Canada

**Keywords:** Bayesian mixed model, genotype-by-environment interactions, phenotypic modeling, remote sensing, seed orchard management

## Abstract

Interior lodgepole pine [*Pinus contorta* Dougl. var. *latifolia* Engelm.], is a keystone species in western Canada and the northwest United States, contributing significantly to natural forest ecosystems and commercial forestry. However, unstable seed yields driven by high conelet (first-year ovulate strobilus) mortality rates (CMR) hinder effective management and breeding efforts in Alberta, Canada. This study explores the potential of different sources of information, such as multispectral imagery, foliar nutrient analysis and microclimatic data, to predict reproductive success across three seed orchards in Alberta. The orchards are designed for deployment at different elevations, were established using different methods (seedling versus grafting), and are different ages. Phenotypic assessments focusing on conelet counts and CMR were conducted in each orchard over a three-year period. Environmental conditions were monitored using HOBO data loggers, and multispectral unmanned aerial vehicle (UAV) imagery (2x/year) was used to provide high-resolution vegetative indices. Foliar nutrient profiles were also analyzed using inductively coupled plasma optical emission spectroscopy (ICP-OES). The datasets were integrated into a Bayesian mixed model framework to identify key predictors that provide genetic and environmental context. Results revealed that genetic factors (clone- or family-based effects) accounted for up to 13% of the phenotypic variance, while environmental contributions ranged from 15% to 27%. Multi-source models improved prediction accuracy in specific scenarios, mainly through genotype-by-predictor interactions. Among all datasets, the fall multispectral imagery showed the greatest potential, with increases of 2% to 6%, in predictive accuracy from the baseline. Predicting the performance of new genotypes remained challenging, highlighting the need for genomic data integration.

## Introduction

Interior lodgepole pine [*Pinus contorta* Dougl. var. *latifolia* Engelm.], is one of the most widely distributed and well-studied tree species in western Canada and the northwest United States (US), valued for its productivity, evolution, biogeographic distribution, and genetic diversity (Thomas et al., 1999; Liewlaksaneeyanawin, 2006; Liewlaksaneeyanawin et al., 2015; Bisbing et al., 2021; Bockstette et al., 2021; Mitchell and Thomas, 2025). In Alberta, lodgepole pine is considered the dominant subclimax species in the subalpine and montane forests and the Foothills Section of the boreal forest (Smithers, 1961). Lodgepole pine is also a keystone species that provides the second-highest harvest volume, after white spruce [*Picea glauca* (Moench) Voss], contributing significantly to commercial forestry in Alberta (FGRMS, 2016). Consequently, forest companies must ensure sufficient cones and seeds for reforestation, particularly with increasing threats from climate change, pests, and pathogens (Bates et al., 2002; Strong, 2006; Sang et al., 2023). However, some lodgepole pine seed orchards in Alberta have failed to meet targeted seed yields by up to 80% for the last decade, primarily due to conelet abortion (Bockstette and Thomas, 2020; Dykstra et al., 2025). Currently, ~17% of pine deployment comes from improved seed in Alberta (Thomas et al., 2024).

Conifer growth cycles are relatively long, and seed yield is often unstable (Ross et al., 2018; Li et al., 2021). Lodgepole pine seeds are frequently held in resin-sealed cones on the tree through serotiny, an adaptive trait in which cone opening is triggered primarily by the intense heat of fire (Lotan, 1976; Bancroft, 1996). Serotiny, and the number of seeds released vary considerably among populations depending on local fire history and location (Pausas, 2015). The ‘fight or flight’ concept (Lauder et al., 2019) proposes that under severe stress, conifers shift resource allocation between growth (‘fight’) and reproduction (‘flight’), with the latter escalating as the likelihood of mortality increases due to stressors such as drought (Andrus et al., 2020). Conelet development and seed yields are affected by a variety of factors, including pollen availability and quality, temperature, light, water availability, and nutrients. Previous work has identified that limiting factors such as low water availability and organic matter loss can affect lodgepole pine health and increase vulnerability against disease (Duan et al., 2015). In another study, sensitivity indicators (pH, C:N, C:P, Ca: Al) have also been related to forest health (Reid et al., 2015). To address these issues, several management techniques, such as topping, pruning, fertilization, hormone treatments, and supplementary pollination, have been developed for improving cone yields (Bockstette and Thomas, 2020; Crain and Cregg, 2018; Li et al., 2021).

Despite the operational significance, conelet mortality rates remain high and largely unexplained. Bockstette and Thomas (2020) reported mortality rates averaging 72%, with some genotypes reaching close to 100%, with significant mortality occurring in the first spring after conelet development, at the time of pollination. Overall, the application of gibberellic acid (GA_4/7_) could not control the ultimate conelet loss, although provenance did play a role in the effectiveness of treatment timing (Bockstette and Thomas, 2020). Another study in British Columbia, found significant temporal correlations between seed production of lodgepole pine trees, from different natural stand seed planning zones (SPZ), and temperature and precipitation (Lew et al., 2017). The same study explained that only three of the 18 SPZs showed a significant trend in annual seed yield because the reproductive capacity in wild stands of lodgepole pine is adapted to decadal-scale climate change.

Given the complexity of conelet mortality, predictive frameworks integrating different sources of information offer a promising alternative for modeling the trait. The use of pedigree relationships to extract breeding values for tree improvement is well established in tree breeding (Grattapaglia and Resende, 2011), and the prediction of phenotypes using dense genomic marker information, known as genomic selection (GS) (Meuwissen et al., 2001; Habier et al., 2007) has seen growing adoption in tree breeding (Grattapaglia et al., 2018; Sharma et al., 2024). The applications of GS in forestry include loblolly pine [*Pinus taeda* L.] (Resende et al., 2012), *Eucalyptus* spp (Duran et al., 2017; Suontama et al., 2019). and *Picea* spp (Beaulieu et al., 2014), and recent studies have suggested using genomic data to manage lodgepole pine (MacLachlan et al., 2017, MacLachlan et al., 2018; Mahony et al., 2020; Ukrainetz and Mansfield, 2020; Azcona et al., 2025). The desire to enhance predictive ability across diverse environments, sites, and years led to the development of models explicitly accounting for genotype-by-environment (G**×**E) interactions (Burgueño et al., 2012; Heslot et al., 2014; Jarquin et al., 2014; Crossa et al., 2017; Fritsche-Neto et al., 2025; Azcona et al., 2025). The use of environmental covariates has been proposed to explain differences in genotypic adaptation driving G**×**E interactions, an approach formalized as ‘enviromics’ (Heslot et al., 2014; Costa-Neto et al., 2021; Resende et al., 2021).

In addition to advancements in statistical modeling, significant contributions have been made in expanding the set of biological traits available as phenotypic predictors (Crossa et al., 2021). Rincent et al. (2018) introduced the concept of phenomic selection, proposing the use of near-infrared spectroscopy (NIRS) to capture secondary traits that serve as surrogates for endophenotypes such as transcripts and metabolites (Mackay et al., 2009). NIRS-based phenomic selection has been applied in crops including wheat [*Triticum aestivum* L.] (Rincent et al., 2018; Lane et al., 2020), soybean [*Glycine max* (L.) Merr] (Zhu et al., 2021), rapeseed [*Brassica napus* L. var. *oleifera*] (Roscher-Ehrig et al., 2024), and sweet corn [Zea mays L.] (Graciano et al., 2025). A methodologically different class of secondary predictors is the unmanned aerial vehicle (UAV) imagery, which differs from NIRS in spectral resolution, spatial scale, and the objectives of its collection (Tayade et al., 2022; Stejskal et al., 2023). UAV imagery has been used to support genetic evaluations (Reynolds et al., 2020) and enhance the predictive accuracy of genomic selection models in crops such as wheat (Jackson et al., 2023; McBreen et al., 2025; Kunwar et al., 2026), maize (Adak et al., 2023), strawberry [*Fragaria* **×** *ananassa* Duchesne] (Sleper et al., 2025), soybean (Van der Laan et al., 2025), and potato [*Solanum tuberosum* L.] (Maggiorelli et al., 2024; Yusuf et al., 2025).

In forest tree species, UAV imagery is of particular interest because it can rapidly quantify plants’ physiological and functional status (Stejskal et al., 2023), with applications in traits related to wood production or disease detection in species such as black poplar [*Populus nigra* L.] (Ludovisi et al., 2017), slash pine [*Pinus elliottii* E.] (Song et al., 2022; Li et al., 2023), and eucalyptus [*Eucalyptus cladocalyx*] (Mora-Poblete et al., 2024). Vegetative indices derived from multispectral UAV imagery have been applied to characterize individual tree attributes in clonal pine seed orchards (Gallardo-Salazar and Pompa-García, 2020) and, particularly in lodgepole pine, UAV color indices have been related to vigor, mortality, foliar disease occurrence, and root disease symptoms in British Columbia plantations (Reid et al., 2016). In this study, we combined three sets of predictors: UAV-based multispectral imagery, foliar nutrient content, and microenvironmental sensor records characterizing local growing conditions to address the following objectives: (i) To assess the contribution of individual and combined sets of variables to the prediction of conelet mortality rate (CMR), initial conelet counts (ICC) and final conelet counts (FCC); (ii) To evaluate the performance of models in different real-life scenarios occurring in lodgepole pine tree improvement programs.

## Materials and methods

### Study site

This study was conducted in three lodgepole pine seed orchards with distinct characteristics, in Alberta, Canada (**Figure 1**). Orchard A is a low-elevation seedling seed orchard (designed for low-elevation deployment) established with family-based seedlings, Orchard B is a high-elevation clonal seed orchard (designed for higher-elevation deployment) established with grafted trees, and Orchard C is a clonal second-generation low-elevation seed orchard established with grafted trees. In this manuscript, Phase 1= first-generation, Phase 2= second generation. All three seed orchards are located at a single site, near Grande Prairie, Alberta, Canada, on land that was previously used for agriculture. The high-elevation lodgepole pine breeding program was initiated in 1977 (John, 2018). The establishment of the clonal seed Orchard B began in 1994 with the planting of trees developed from scion material collected from 34 parental genotypes, grafted onto 3-year-old seedling rootstock, for a total of 174 trees (Bockstette and Thomas, 2020). Additional genotypes were added to the orchard until 2013, for a total of 110 genotypes and a total of 1550 trees. The low-elevation lodgepole pine breeding program was initiated in 1976 (John, 2010). The establishment of the phase 1 (Orchard A) seedling seed orchard began in 1986 and it was rogued in 1993 (from 16,500 to 8,354 trees) and in 1999-2000 (from 8,354 to 2,886) (John, 2010), with a current total of 87 families. The establishment of the phase 2 clonal seed Orchard C began in 2005, with 113 clones and 1,242 trees (John, 2010). The orchards represent distinct elevation classes corresponding to different breeding zones within Alberta’s lodgepole pine program. Orchard A and Orchard C are low-elevation sites (~600–700 m a.s.l.), whereas Orchard B is located at higher elevation (~900–1,100 m a.s.l.). There is no genetic or physical connectivity between Orchard A (1.5-generation seedling orchard) and Orchard C (second-generation clonal orchard); populations were analyzed as independent due to the absence of pedigree or genomic links. Apparent connectivity signals are more likely driven by within-orchard covariance and spatial or environmental effects, including spacing and orchard design. The orchards were established operationally using randomized unreplicated seed orchard layouts, which are best modeled as completely randomized designs (CRD) without blocking, a common approach in seed orchards where replication occurs at the genotype level rather than via formal experimental blocks. Because sampling was distributed across orchards rather than clustered, large-scale environmental clustering effects were minimized. However, as in all operational orchards, some residual spatial autocorrelation cannot be fully excluded, which may contribute to environmental variance components observed in the models.

**Figure 1:**
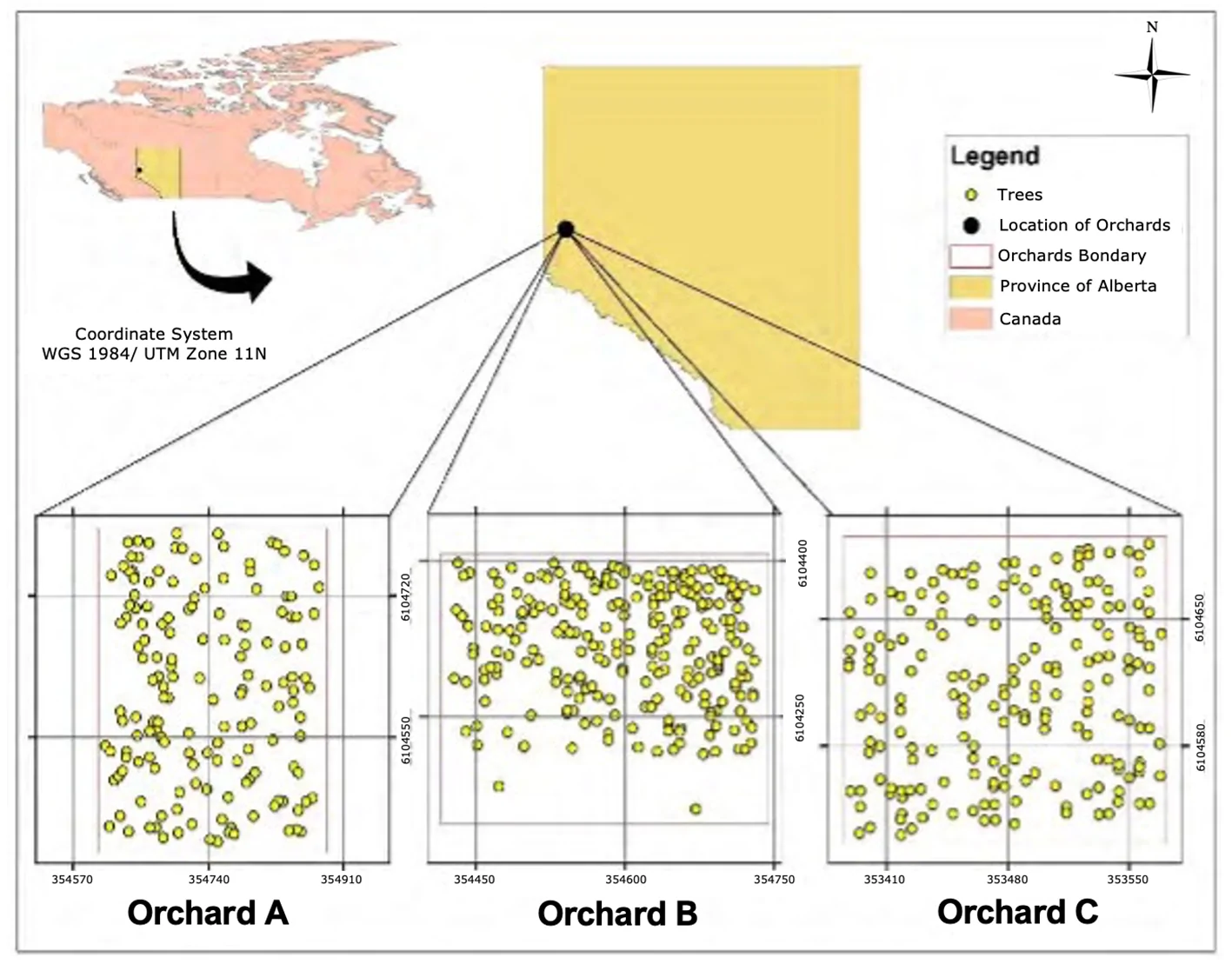
Location of the three lodgepole pine orchards and the location of each tree within each orchard under study in Alberta, Canada.

### Phenotyping: sampling of conelet mortality

Conelet mortality was collected across 593 total trees, averaging six replicates per family or clone within each orchard. This included 26 half-sib families (156 trees) in Orchard A, 33 clones (197 trees) in Orchard B, and 40 clones (240 trees) in Orchard C (**Table 1**). For each tree, three randomly selected branches in the middle and upper part of the crown were labeled using flagging tape, and the conelets of each labeled branch were counted in June (initial emergence) and early August (after conelet mortality occurs, **Figure 2**). Ladders were used to reach the branches and count the conelets from the flagging tape to the end of each branch in Orchards B and C, while a gas-powered lift was used to reach the branches in Orchard A. This procedure was used from 2014 to 2021 in Orchard B and from 2019 to 2021 in the remaining two orchards (**Table 1**, **Figure 1**).

**Table 1:** Description of the three lodgepole pine seed orchards including their code, number of target trees assessed, type of seed orchard, approximate age of orchard, number of families or clones and available phenotypes by year and percent (%) of missing values in brackets.

					Number of unique		Available phenotypes (% missing values)		
Orchard code	Elevation (m.a.s.l.)	Number of trees	Orchard type	Age	Half-sib families	Clones	2019	2020	2021
A	600-700	156	seedling	36	26	0	155 (0.6%)	24 (84.6%)	156 (0%)
B	900-1100	197	grafted	28	0	33	194 (1.5%)	58 (70.5%)	196 (0.5%)
C	600-700	240	grafted	17	0	40	237 (1.2%)	44 (80%)	236 (1.7%)

**Figure 2:**
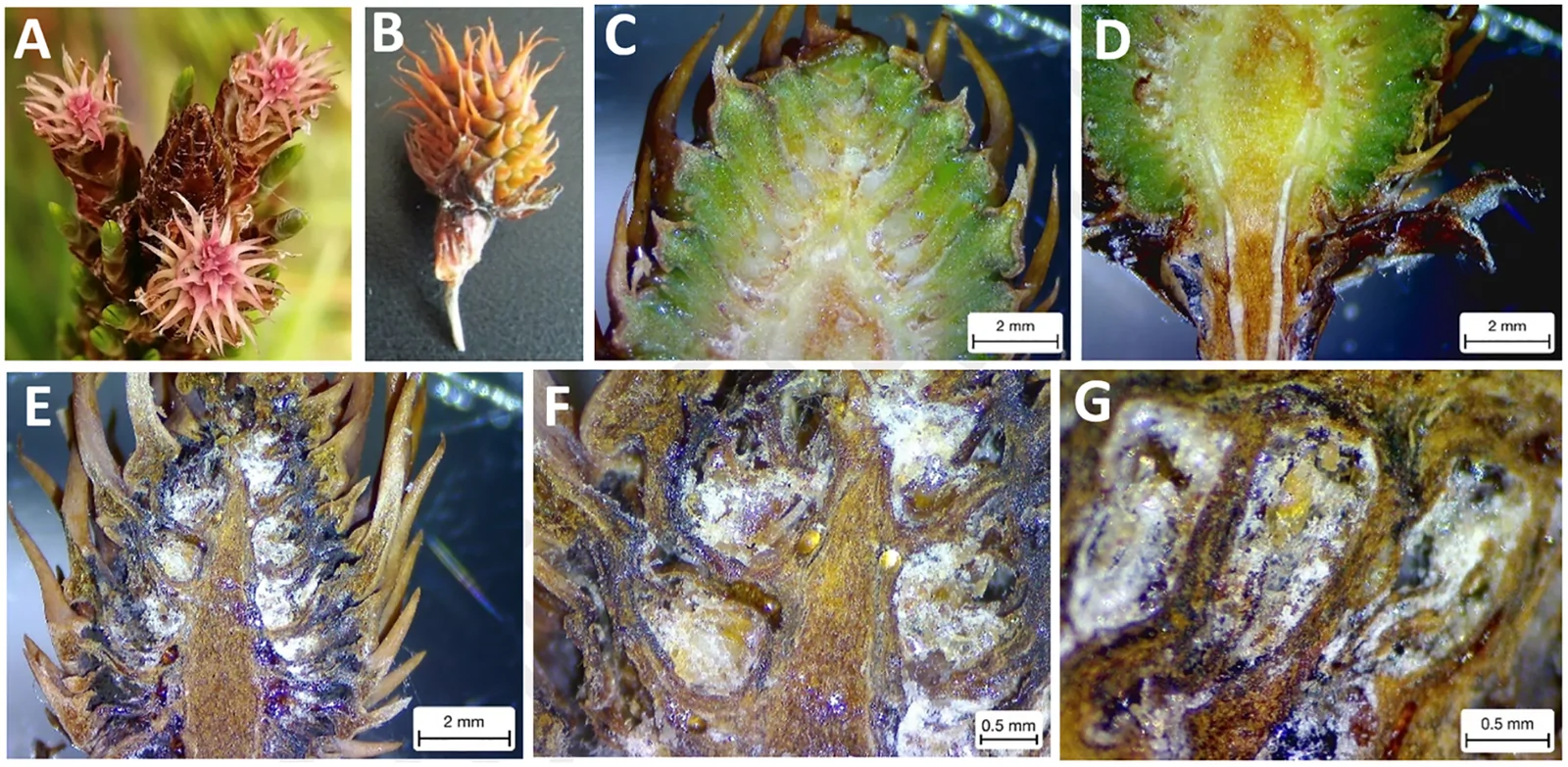
Visualization of aborted and healthy lodgepole pine conelets. **(A–G)** Conelet dissection using a blade at approximately three weeks post receptivity. **(A)** Healthy conelet in the stage of pollen reception. **(B)** Healthy conelet with successful pollination. **(C)** Conelet with healthy scales, bracts, and pollinated ovules. **(D)** Conelet with healthy peduncle. **(E)** Conelet showing browning (mortality), starting in the peduncle, and spreading into the conelet. **(F, G)** Cavities with aborted ovules and necrosis.

### Collection of predictor datasets

For foliar nutrient content, needles were collected from all 593 trees under study in 2019, 2020, and 2021. Immediately after collection, needle samples (~1 g fresh weight per tree) were placed into labelled paper coin envelopes and placed into a cooler, after which they were returned to the University of Alberta for processing. Needles tissue was then dried at 65 °C for three days, then ground using a ball grinder (Qiagen TissueLyser II; 20 mm stainless-steel grinding ball, 30 Hz for 1 min). Ground tissue was then shipped to Alberta Innovates for sample analysis.

The Inductively Coupled Plasma-Optical Emission Spectroscopy (ICP-OES), using a Thermo iCAP6300 Duo (N. America) inductively coupled plasma-optical emission spectrometer (ICP-OES) (Thermo Fisher Corp., Cambridge, United Kingdom) was used to determine the N, P, K, Ca, Mg, S, B, Cu, Zn content in needles at the Natural Resources Analytical Laboratory (NRAL), Faculty of Agricultural, Life & Environmental Sciences, University of Alberta, Canada.

For microenvironment (microclimate) measurements in 2019, 12 data loggers (HOBO Data Logger, MX2301A model) were installed in the three pine seed orchards: four sensors in Orchard A, four sensors in Orchard B, and four sensors in Orchard C (only three recorded). In 2020, the number of sensors was increased to 12 in each orchard and systematically distributed throughout each orchard. In 2021, only data from 24 sensors corresponding to Orchards B and C were collected (**Supplementary Table 1**). For the three years of study, temperature (average, maximum, minimum) and relative humidity (average, maximum, minimum) were recorded using data from June, July, and August of each year, corresponding to the same time of year when conelet mortality rates and foliar nutrients were measured.

For multispectral values, in the last week of May (considered in this work as Spring), and the first week of August (considered in this work as Fall), of 2019, 2020, and 2021, Progressive Media Video company flew the three lodgepole pine orchards (A, B and C) and obtain the multispectral UAV images using a DJI Phantom 4 Multispectral (P4M) quadcopter. The P4M camera has a total of six imaging sensors, five multispectral sensors (i.e., blue, green, red, red-edge, and near-infrared bands), and one red-green-blue (RGB) sensor, all with a global 2 MP shutter. The P4M operates with direct onboard georeferencing, which means it has a global positioning system (GPS) that geotags the coordinates of each acquired image. This tagging indicates that the GPS capability directly georeferenced the acquired images during the flight mission. Vegetation indices were derived from orthomosaic reflectance data following individual tree segmentation. For each tree crown, reflectance values were extracted and summarized using central tendency metrics (mean pixel values) within the segmented canopy area, a standard approach in UAV-based phenotyping to reduce pixel-level noise. Flight height was 60 m above ground level, corresponding to spatial resolutions of approximately 3 cm per pixel, which is sufficient for individual tree crown delineation in orchards (consistent with UAV forestry applications). No additional pixel filtering (e.g., shadow masking beyond segmentation) was applied; thus, extracted values represent the aggregate spectral signal of each crown segment. (**Figure 2**). All procedures were performed in ArcGIS 10.7.1. Since vegetation indices are proxies of vegetation vigor or productive status (**Supplementary Table 2**), we tested multispectral vegetation indices recorded from the UAV in the study area (Gallardo-Salazar and Pompa-García, 2020).

### Statistical analysis

The predictive pipeline in this study was implemented using CHiDO (González et al., 2025) in R (R Development Core Team, 2024). Custom scripts were used to preprocess the input data and postprocess the results. The detailed methodology is described in the following subsections. The analysis was conducted independently for three conelet-related traits: initial conelet count (ICC), final conelet count (FCC) and conelet mortality rate (CMR), calculated as the difference between initial and final counts relative to the initial value.

### Pre-processing steps for predictor data

The phenotypic data file was structured so that each observation was identified by three distinct linking identifiers, each enabling its connection with the corresponding predictor datasets. The first one is the genetic identifier (GID), shared by all replications within the same genetic group: either half-sib families in the seedling Orchard A or ramets of the same clone in the graft Orchards B and C. The second is the environment identifier (EID), representing combinations of Orchards A, B and C and years (2019, 2020 and 2021), yielding nine environments (e.g., A.2019 or C.2021) and connecting the phenotypic file with the microenvironment data. The third is the unit identifier (UID), which uniquely identifies each phenotypic observation as a combination of an individual tree (1 to 593) and the observation year, enabling its linkage to foliar nutrient and multispectral measurements (**Table 2**).

**Table 2:** Multi-source datasets used in this study.

Predictor	Factor	Term	# of levels	# of features	% missing
Microenvironment	Environment	M	9	45	11.11
Nutrients	Unit	N	1300	12	14.52
Spring-Multispectral	Unit	S	1300	17	0.23
Fall-Multispectral	Unit	F	1300	17	0.95

Factor denotes the element that is characterized by each predictor dataset. Each factor has a specific number (#) of levels and a variable number of features. The term of the predictor is the letter that will be used to denote them in models. The percentage (%) of missing values across datasets is also reported.

### Modeling

In this study, CHiDO (González et al., 2025) was used to apply mixed linear models within a Bayesian framework implemented via the BGLR package (Pérez and de Los Campos, 2014). The general model can be defined as y=μ+η+ϵ, where y is the vector of responses, μ is a common intercept, η is the linear predictor and ϵ is the residual term. The linear predictor is defined as the sum of t random effects such that $\eta =\sum_{i=1}^{t}u_{i}$ and $u_{i}∼MVN(0,\;K_{u_{i}}\sigma_{u_{i}}^{2})$, where $K_{u_{i}}$ is the user-defined covariance structure for the *i*^th^ term and $\sigma_{u_{i}}^{2}$ is the scaling variance. This general model can incorporate multi-source information, either as a main effect or as part of an interaction between two or more terms, as described by Jarquín et al. (2014) and implemented in CHiDO. All models were trained using 40,000 total MCMC iterations, 2,000 burn-in iterations, and a thinning factor of 5, resulting in 7,600 posterior samples per chain. The default BGLR prior hyperparameters were used. Chain convergence was assessed using the Geweke diagnostic (Geweke, 1991) implemented in the coda R package (Plummer et al., 2006).

CHiDO allows users to introduce uninformative terms for the environment (‘E’) and genetic (‘L’) effects, so that the different levels within each factor are considered independent. These terms are advantageous when other predictors are unavailable but are limited when non-null covariance between levels is expected. In this study, ‘E’ and ‘L’ are used to assume independence among levels of the environment (EID) and genetic (GID) factors, respectively. The term ‘L’ comes from ‘line’, an umbrella term for genotypes or varieties evaluated in the field when pedigree or genomic data are unavailable. The microenvironment information, ‘M’, is used to break the assumption of independence between environments. The nutrient ‘N’, spring multispectral ‘S’, and fall multispectral ‘F’ data are used to break the assumption of independence among tree-level observations.

The following models were utilized by defining different linear predictors with both main and interactive terms:

M0: E + L + EL

M1: E + M + N + S + F

M2: E + L + EL + M + N + S + F

M3: E + L + EL + M + ML

M4: E + L + EL + N + NL

M5: E + L + EL + S + SL

M6: E + L + EL + F + FL

M7: E + L + EL + M + N + S + F + ML + NL + SL + FL

Two-letter terms denote interaction effects between two components. M0 is a baseline model that assumes independence among environment and genetic levels. M1 is a predictors-only main effects model that ignores the genetic background of the trees. M2 extends M0 by incorporating all the predictors main effects in M1. Models M3-M6 are extensions of M0 that evaluate a single set of predictors (‘M’, ‘N’, ‘S’, ‘F’, respectively) and its interaction with the genetic background term (e.g., ‘ML’). M7 is the full model, combining all main and interaction effects.

### Cross-validation

Three widely used cross-validation scenarios in multi-environment trials (METs) for plant breeding, as described by Crossa et al. (2017), were tested. All models were evaluated using 5-fold cross-validation, with 20% of the observations masked as missing (test set) and 80% used for training. A total of 10 replications were conducted to reduce the variability due to random data partitioning.

#### CV2: random cross-validation

In this scheme, random observations are removed from the phenotypic dataset and used as the test set. This approach simulates sparse testing scenarios in which only a subset of trees is phenotyped and the rest are predicted, potentially reducing phenotyping costs.

#### CV1: predicting new genotypes

Here, all phenotypic observations for a group of genotypes are excluded from the dataset and used as the test set. This approach tests the model’s ability to predict genotype performance without phenotypic records when other trees with similar genetic backgrounds are unavailable.

#### CV0: predicting new environments

This scheme simulates predicting an unknown location or evaluating the performance of a known location under different conditions (such as in future or past seasons or years). The CV0 scheme evaluates the model’s ability to generalize across environments.

### Performance metrics

Three different metrics were used to evaluate model performance. First, variance components ( $\sigma_{u_{i}}^{2}$) were calculated by fitting the model to the entire dataset, with each component representing the importance of the term to the target trait variation. Next, the deviation between observed and predicted values obtained through cross-validation was quantified using Pearson’s correlation and Root Mean Squared Error (RMSE). Although correlation provides a measure of how well the models rank the observations, it does not quantify the absolute deviation from the observed values, which can be of interest in traits such as those related to time (Jarquin et al., 2020). Therefore, RMSE is also used here. For this study, correlations were calculated separately for each of the environments and then averaged using the number of observations as the weighting factor to prevent correlation inflation due to a cross-environment variation (Tiezzi et al., 2017). Additionally, to assess the ability of the models to approximate the average trait value for an environment never observed during training (i.e., CV0 tasks), the predicted value for each environment was averaged across replications in which it appeared in the test set and compared to the observed environment mean using RMSE. The number of environments in which each model achieved the lowest absolute deviation from the observed mean was also recorded.

## Results

### Variance components

The average environmental contribution across models was 14.9% for ICC, 20% for FCC, and 26.4% for CMR (See **Supplementary Table 3** for all variance components). The residual unexplained variance in the predictors-only model M1 (82.1% for ICC, 77.4% for FCC, and 71.3% for CMR) was substantially higher than in the genetic baseline M0 (68.9%, 64.9%, and 58.6%, respectively). The model with the most significant reduction in residual variance was consistently the full model M7, with values of 60.4% for ICC, 56.7% for FCC, and 50.5% for CMR.

The genetic background term ‘L’ contributed 12.5% to ICC, 11.9% to FCC and 10.6% to CMR in M0, and remained stable across models M2 to M5 but dropped to an average of 9.7% in ICC, 9.7% in FCC, and 8.5% in CMR in models M6 and M7, confirming that the ‘FL’ interaction between the fall multispectral data and genetic background absorbed variance previously attributed to the main genetic effect ‘L’ (**Figure 3**). The genotype-by-environment interaction term ‘EL’ declined consistently across all models incorporating interactions, from 3.9%, 3.5%, and 4.0% in M0 to 1.0%, 1.0%, and 1.3% in M7 for ICC, FCC, and CMR, respectively. Notably, EL also decreased in M2, suggesting that predictors main effects partially absorb interactions between genetic background and environment.

**Figure 3:**
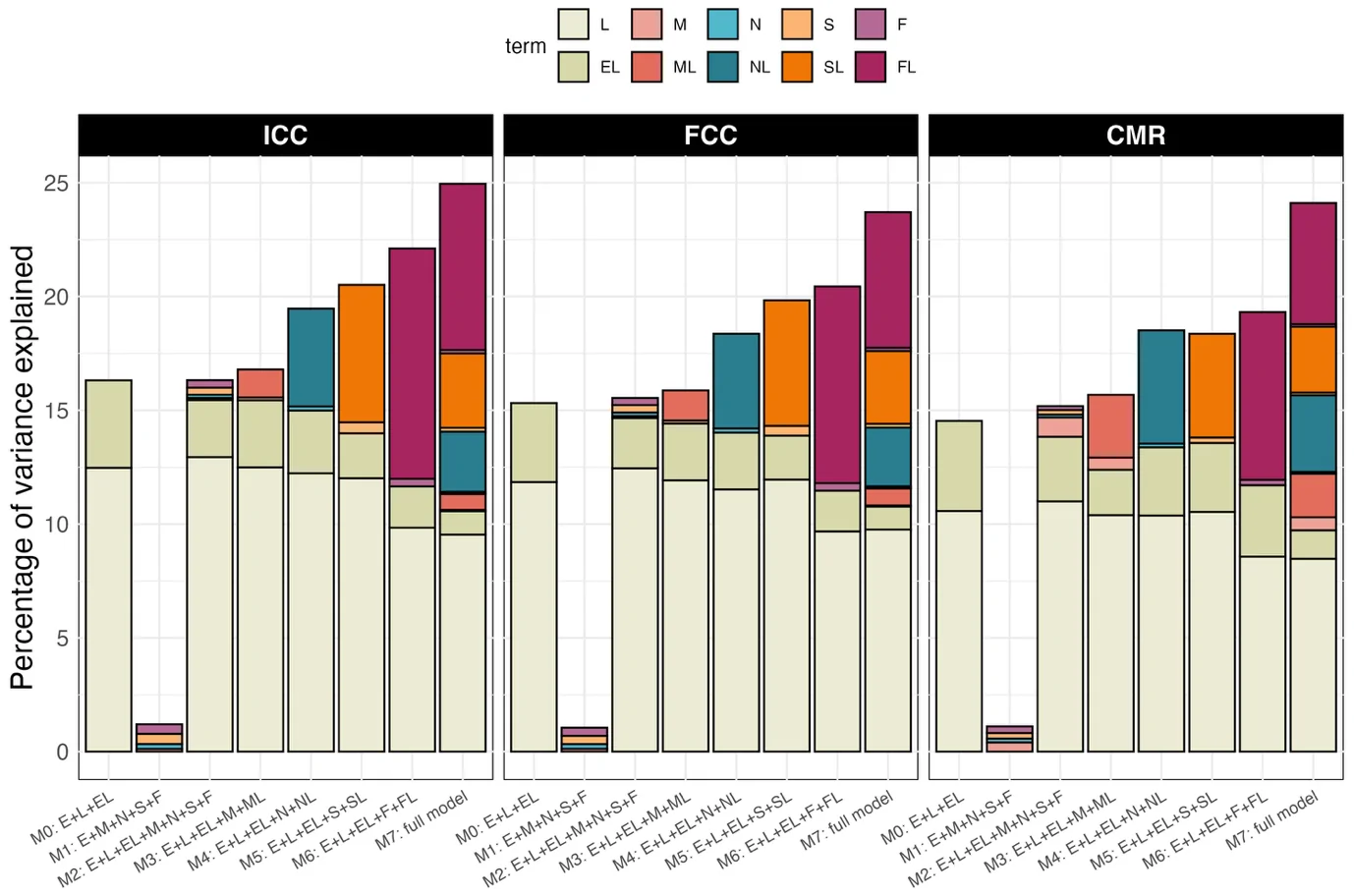
Variance components in the lodgepole dataset. The percentage of phenotypic variance explained by each term and model. The R (residual) and E (environment, year-location combination) terms have been omitted to stress the contribution of each predictor dataset. Each panel shows one trait, ICC (Initial Conelet Count), FCC (Final Conelet Count), and CMR (Conelet Mortality Rate), respectively. The x-axis denotes the models, and the colors represent the term. The terms shown are E (environment), N (nutrients), S (multispectral spring), F (multispectral fall), M (microenvironment), L (genetic background). The two-letter terms represent interactions (e.g., NL is nutrients x genotype interaction). M7: full model is E+L+EL+M+N+S+F+ML+NL+SL+FL.

Predictors main effects were negligible across models, ranging from 0.1% to 0.8% across traits and models. The microenvironment dataset main and interactive terms were relatively more important for CMR, as shown in **Figure 3**. In M7, the four predictor-by-genotype interactions combined explained 14.1%, 12.5%, and 13.6% of variance for ICC, FCC and CMR, respectively, though their individual contributions were reduced relative to single-interaction models M3-M6 (**Figure 3**), suggesting partial overlap in the variance captured across predictors layers. Nevertheless, the combined genetic-related variance (‘L’ + ‘EL’ + all other interactions) in M7 reached 24.7%, 23.3% and 23.3% for ICC, FCC, and CMR, compared to 16.3%, 15.3% and 14.5% in M0, highlighting the added value of predictors-by-genotype interactions beyond the baseline genotype-by-environment term.

### Predictive performance

Genetic models (M0, M2-M7) consistently outperformed the predictors-only model M1 in both the CV2 and CV0 scenarios (**Figure 4A**). In CV2, M1 showed near-zero or negative predictive accuracy across traits (r = 0.05, 0.01, and -0.1 for ICC, FCC and CMR, respectively), while genetic models ranged from r = 0.36-0.38 for ICC, r = 0.38-0.40 for FCC, and 0.32-0.33 for CMR. The baseline model M0 achieved r = 0.361, 0.380 and 0.324 for ICC, FCC and CMR, respectively. Among models with predictors terms, M6 (E+L+EL+F+FL) obtained the best accuracy for ICC (r = 0.382) and CMR (r = 0.334), while M2 (E+L+EL+M+N+S+F) showed a better performance for FCC (r = 0.398). Notably, M6, which includes only fall multispectral imagery and its genotype interaction, matched or exceeded the full model M7 across all traits (**Figure 4A**), suggesting that fall multispectral data captured the most relevant signal. In terms of RMSE, genetic models were largely comparable to each other, with average values of 11.3, 9.5 and 21.0 for ICC, FCC and CMR, respectively, while M1 showed substantially larger errors across traits (**Figure 4B**). Overall RMSE increased progressively across cross-validation scenarios (CV2 < CV1 < CV0).

**Figure 4:**
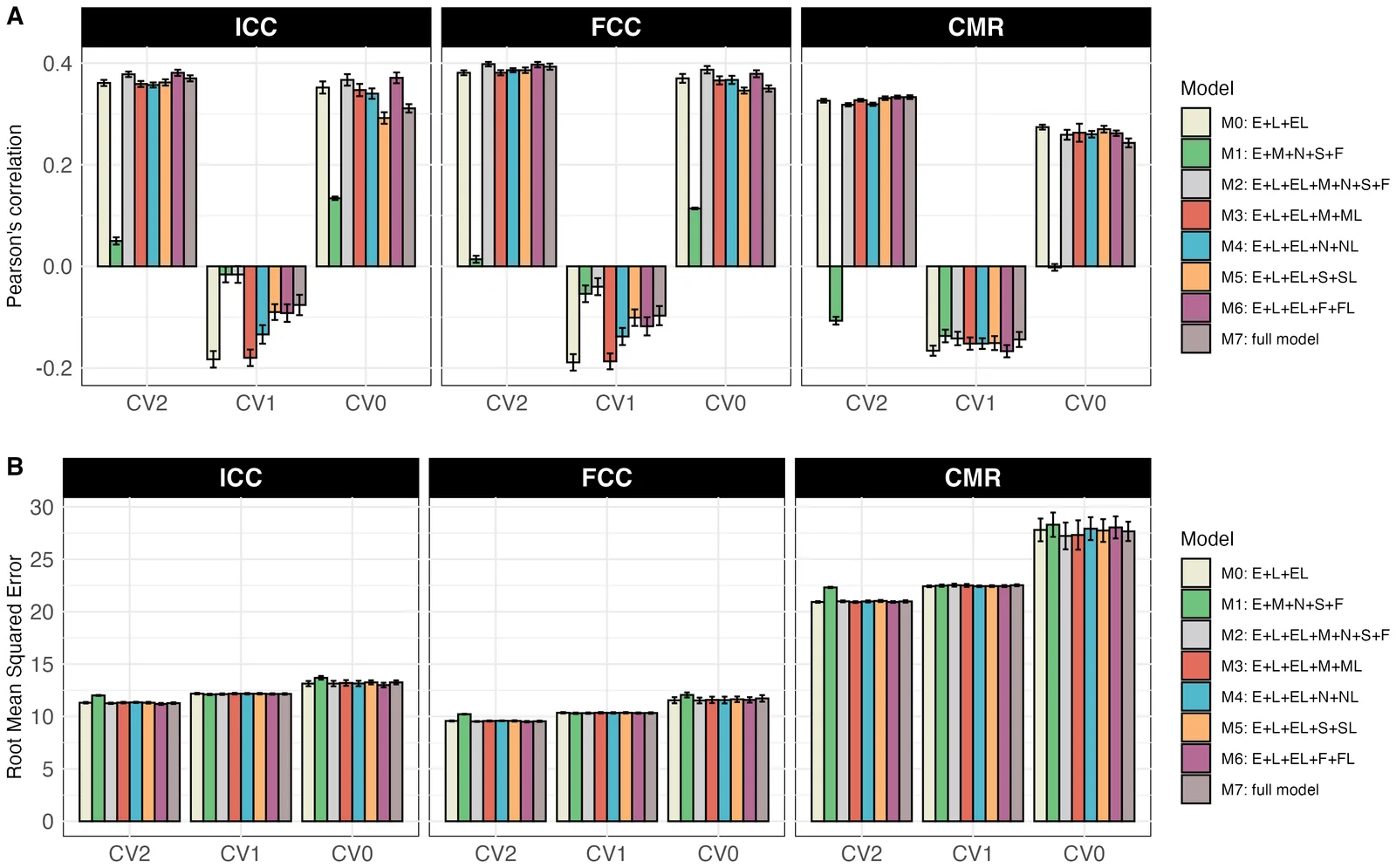
Performance of models. The y-axis represents the accuracy as Pearson’s correlation between observed and predicted values **(A)** and the root mean squared error **(B)**. The higher the correlation and the lower the RMSE, the better. The x-axis represents the cross-validation scenario where CV2 is sparse testing, CV1 is predicting new clones in seen environments and CV0 is predicting seen clones in new environments. Each bar denotes a model that is identified with a color. Each panel shows one trait, ICC (Initial Conelet Count), FCC (Final Conelet Count), and CMR (Conelet Mortality Rate), respectively. The terms used in the models are E (environment, year-location combination), N (nutrients), S (multispectral spring), F (multispectral fall), M (microenvironment), L (genetic background). The two-letter terms represent interactions (e.g., NL is nutrients x genotype interaction). M7: full model is E+L+EL+M+N+S+F+ML+NL+SL+FL.

In the CV0 scenario, overall predictive accuracy was slightly lower than in CV2, with best-model correlations of rM6 = 0.371 for ICC, rM2 = 0.385 for FCC, and rM0 = 0.273 for CMR, the only trait-CV combination in which the baseline outperformed all other models. At the environment level, CV0 best-model correlations were entirely positive, ranging from r_M1_ = 0.095 (CMR, A.2019) to r_M6_ = 0.749 (FCC, C.2020), while CV2 ranges included negative values in several environments (**Figure 5A**). Overall, 16 out of 27 trait-environment combinations achieved higher accuracy in CV0 than in CV2, with the highest single-environment accuracies recorded in environment C.2020, where M6 achieved r = 0.532 and r = 0.749 for ICC and FCC, respectively. Another notable result is that neither ICC nor FCC could be predicted in environment A.2020 under the CV2 scenario, contrasting with the CV0 scenario, where model M1 yielded the highest accuracy, r = 0.233 and r = 0.300, respectively (**Figure 5A**). Finally, no model achieved positive overall predictive accuracy for any trait under the CV1 scenario (**Figure 4A**). The only exceptions were found at the environment level (C.2019) where M1, again, could predict ICC and FCC with accuracies r = 0.207 and r = 0.125, respectively.

**Figure 5:**
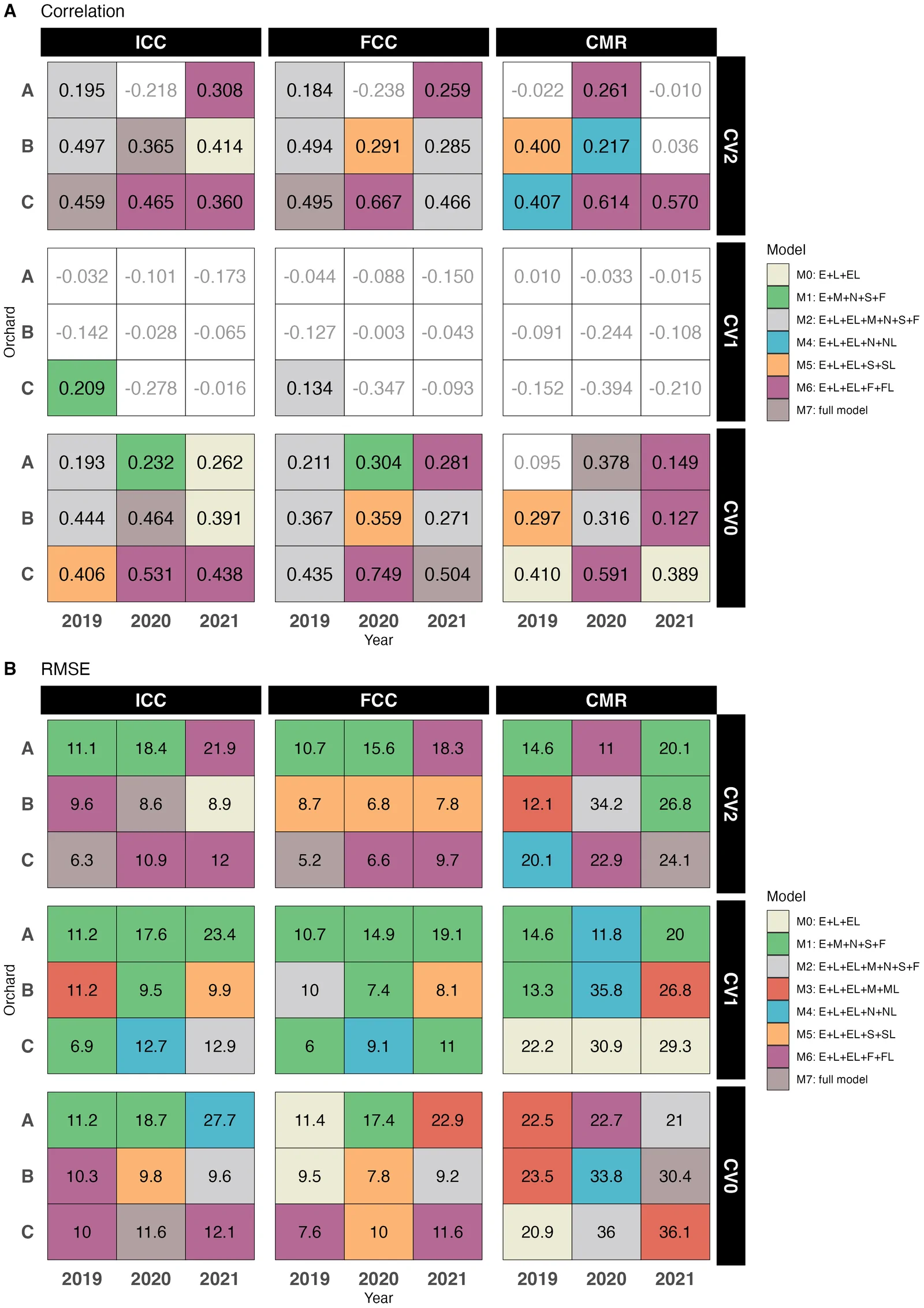
Best performing model in each environment and trait. This figure is an extension of **Figure 4**, where the average values across environments were reported. There are nine panels in a 3x3 grid, where columns represent traits (ICC (Initial Conelet Count), FCC (Final Conelet Count), and CMR (Conelet Mortality Rate), where columns represent the year or season, and the rows represent and rows are cross-validation scenarios. Inside each panel is a 3x3 sub-gr the orchard in which the data was collected. The number inside the grids represents the accuracy **(A)** and RMSE **(B)** obtained by the best model, which is represented by the color. Regarding accuracy, when it was lower than r = 0.10, none of the models were considered to deliver a meaningful prediction. The terms used in the models are E (environment, year-location combination), N (nutrients), S (multispectral spring), F (multispectral fall), M (microenvironment), L (genetic background). The two-letter terms represent interactions (e.g., NL is nutrients x genotype interaction). M7: full model is E+L+EL+M+N+S+F+ML+NL+SL+FL.

### Prediction of environmental intercepts

In the CV0 scenarios, all observations belonging to a specific environment are predicted, resulting in the training set lacking information about that particular environment. The observed and predicted environmental means were calculated to assess the models’ ability to approximate the intercepts of unseen target environments (**Figure 6**, **Table 3**). As anticipated, models tended to predict values that averaged the overall mean of the training population, which made it challenging to approximate unseen environments with extreme intercepts. However, certain models demonstrated superior performance in specific instances (**Figure 6**). For ICC, the baseline model M0 achieved the lowest RMSE (7.09), although all models remained within roughly 0.6 RMSE units of one another, with M0 and M2 most frequently the best-performing model in a single environment (two of nine each, **Table 3**). A similar pattern was found for FCC, where M5 had the lowest overall RMSE (7.24), but no model was best in more than two of nine environments. Finally, M2 achieved the lowest overall RMSE (17.74) in CMR, followed by M3 (17.75), which was the model most frequently best in a single environment (three of nine).

**Figure 6:**
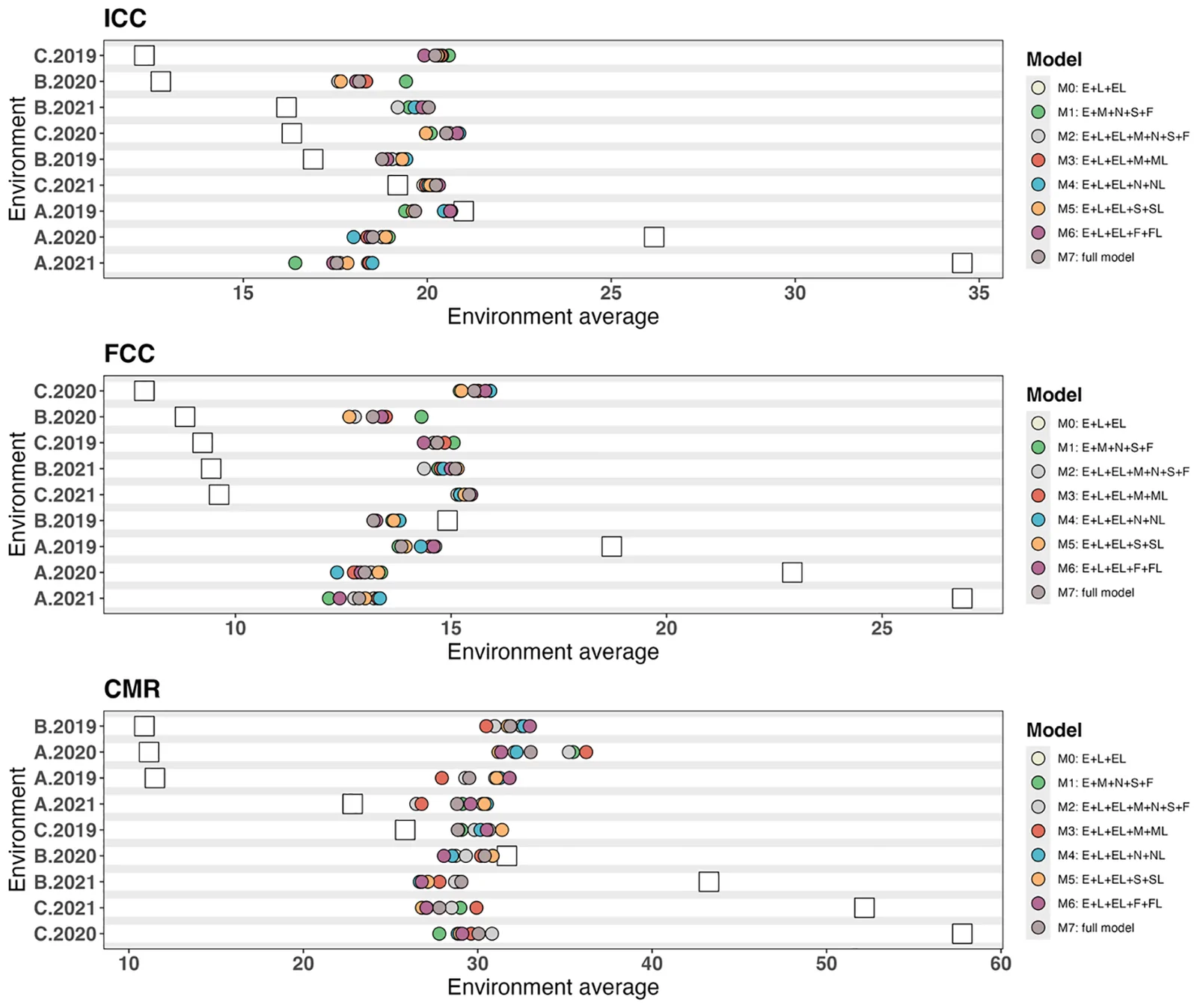
Observed and predicted environmental means in CV0. Each grid represents a trait. Environments are defined on the y-axis as a combination of an orchard code and a phenotyping year, and sorted by increasing environment average, shown on the x-axis. The white squares represent the observed average of each environment, while the circles represent the average predicted value in the CV0 cross-validation scheme, with colors denoting models. M7: full model is E+L+EL+M+N+S+F+ML+NL+SL+FL.

**Table 3:** Prediction of environment intercepts under the CV0 scenario.

	RMSE			Best in n environments		
Model	ICC	FCC	CMR	ICC	FCC	CMR
M0: E+L+EL	**7.09**	7.29	18.58	2	1	0
M1: E+M+N+S+F	7.66	7.52	18.67	1	2	1
M2: E+L+EL+M+N+S+F	7.17	7.28	**17.74**	2	2	2
M3: E+L+EL+M+ML	7.10	7.29	17.75	0	0	3
M4: E+L+EL+N+NL	7.12	7.38	18.68	1	2	0
M5: E+L+EL+S+SL	7.12	**7.24**	18.38	1	1	1
M6: E+L+EL+F+FL	7.28	7.48	18.60	1	1	0
M7: full model	7.29	7.41	17.81	1	0	2

For each trait (ICC, FCC, CMR), the RMSE between predicted and observed environment means is reported across all nine environments. Best performance in specific environments denotes the number of environments (out of nine) in which each model achieved the lowest RMSE for that trait. The terms used in the models are E (environment, year-location combination), N (nutrients), S (multispectral spring), F (multispectral fall), M (microenvironment), L (genetic background). The two-letter terms represent interactions (e.g., NL is nutrients x genotype interaction). M7: full model is E+L+EL+M+N+S+F+ML+NL+SL+FL.

## Discussion

This study explored the potential use of tree- and environment-level predictor datasets to capture variation patterns in three lodgepole pine orchards for conelet-related retention traits and attempted to predict non-phenotyped trees across different locations and seasons. A Bayesian mixed-model approach (Jarquin et al., 2014; Pérezde Los Campos, 2014; González et al., 2025) was used, benchmarking model performance using standard tools from multi-environment trials (METs) in plant breeding: Pearson’s correlation as a measure of accuracy and RMSE as an indicator of precision. Three well-known cross-validation scenarios (CV2, CV1, and CV0) (Crossa et al., 2017; Lane et al., 2020; McBreen et al., 2025) were applied to mimic distinct research questions and operational practices. Summary metrics were computed at the environment level to avoid environment-related confounding factors from inflating accuracy values, which could misrepresent insights and conclusions.

### The role of the genetic background term (‘L’)

When considering phenotypic prediction in tree breeding, GS is often preferred in plant breeding due to its ability to leverage genetic relationships between individuals (Grattapaglia and Resende, 2011; Resende et al., 2012). However, neither pedigree nor genomic marker information was available in this study, and the only genetic information at our disposal was the ‘L’ term, which connected observations of clones from the same genotype in grafted orchards, and half-sib families, in the seedling orchard. It is important to note that this mechanism enables a within-group phenotyping average that is fundamentally different from genomic prediction, where kinship matrices allow information to flow between related, but not identical genotypes. The predictive accuracy observed in CV2 and CV0 should therefore be understood as driven by phenotyping average of observed families. It is worth noting that the observed CV2 accuracies are consistent with the theoretical ceiling imposed by the proportion of variance attributed to the genetic background.

Predictor main effects with the ability to connect trees from different families (‘N’, ‘S’, ‘F’) were largely redundant with each other and with the variance already captured by ‘L’, consistent with the co-variation between foliar chemistry and spectral reflectance reported in other forest species (Reid et al., 2015; Gallardo-Salazar and Pompa-García, 2020) and confirming that repeatability across seasons drives prediction. Nevertheless, the ‘L’ term and its interactions with predictor datasets consistently explained more variance and delivered higher predictive accuracy than predictors main effects alone across all traits. These results are broadly aligned with the literature demonstrating that combining genomic and phenomic datasets improves prediction (McBreen et al., 2025; Roscher-Ehrig et al., 2024; Zhu et al., 2021; Jackson et al., 2023), although the absence of genomic information in this study likely constrains the interaction-level contributions of the predictor terms relative to what could be achieved with proper kinship matrices.

Among the interaction terms, fall multispectral imagery was the most relevant contributor: integrating ‘FL’ with the genetic background (M6) improved upon M0 for ICC (~6%) and FCC (~4%), while matching or exceeding the full model M7 for both traits, suggesting that genetic backgrounds differ in how their fall canopy status responds across environments. These results are comparable to those by Jackson et al. (2023), where genomic and hyperspectral phenomic information increased models’ predictive ability in a multi-environment setting by 6% to 12%. The results for CMR differed from the count traits: while the ‘FL’ interaction provided a ~2% improvement over the baseline in CV2, the CMR-CV0 combination was the only trait-scenario combination where no model outperformed the baseline. This suggests that in the absence of observations from the target environments in the training set, the best strategy for CMR prediction is to simply average across other seasons and correct for genotype-by-environment (‘EL’) deviations. The fall multispectral imagery signal was strongest for ICC and progressively weaker for FCC and CMR, suggesting that canopy status in early August is more related to the tree’s overall photosynthetic capacity, which determines how many conelets are initiated, rather than the specific rate of mortality thereafter.

### Predictor datasets as genetic proxies - the CV1 case

The systematic inability of models to achieve positive overall predictive accuracy under the CV1 scenario is a consequence of their reliance on phenotypic observations of other trees from the same genetic background or even the same tree in another season. In this situation, the ‘L’ term is redundant by definition and penalizes models relying on it. We anticipated the slightly negative correlation of the baseline model (partitions with higher-than-average training set will keep lower-than-average test sets, and vice versa) but expected the multi-source datasets to help connecting information between non-directly related trees.

The predictors-only model M1, containing no ‘L’-related terms was comparatively least affected: although it remained negative in the overall accuracy, it achieved among the highest correlations when predicting ICC and FCC in C.2019, which happens to be the orchard with the largest number of phenotyped trees (n = 237, **Table 2**), tied with M2 for ICC, and surpassed by M2 for FCC. M7, also carrying all the predictors main effects, obtained positive but lower accuracy in this environment. Another particularity of M1 was that it yielded the lowest RMSE in most of the environments under the CV1 scenario for ICC and FCC (**Figure 5B**). These results lead to a clear and practically important conclusion: multi-source data alone could not replace genomic marker information for relating trees and hence predicting the performance of unobserved genetic backgrounds. Multi-source datasets should be considered a complement to, not a substitute for, genomic information in operational seed orchard management under a CV1-like scenario. It is worth noting that Rincent et al. (2018), who proposed NIRS-based phenomic selection in wheat and poplar, used tissue-chemistry reflectance as endophenotypic regressors in a proof-of-concept which is both methodologically and biologically different that the case studied here. Incorporating pedigree or genomic information remains the most direct path to improved CV1 accuracy (Roscher-Ehrig et al., 2024; McBreen et al., 2025).

### Less is more – parsimony and overfitting across CV scenarios

Having multiple replicates of the same genetic background in a given environment makes CV2 theoretically easier for models because: (i) the target environment distribution is partially known through estimates of the sample mean and variance using training data, which explains why the RMSE in CV2 and CV1 is lower than in CV0 (**Figures 5A, B**), and (ii) the tree’s genetic background has been observed in other trees in the same target environment. Our results showed, however, that predictive accuracy, i.e., the ability to rank genotypes based on their phenotypic performance in a given environment, was comparable in both CV2 and CV0. In fact, CV0 accuracies were higher for the latter in a majority of environment-trait combinations (**Figure 5A**), contrasting with studies using genomic information where CV0 consistently falls below CV2 (McBreen et al., 2025; Lane et al., 2020).

A notable example of this phenomenon is environment A.2020, where ICC and FCC traits could not be predicted under the CV2 scenario but where M1, the main effects only model, could predict under CV0. As for CMR, the model integrating fall multispectral data, M6, was the best predictor in both CV2 and CV0, with a 44% increase in the latter (**Figure 5A**). However, this specific result should be interpreted cautiously, as it could be argued that it is due to the smaller sample size in A.2020 (n = 24). Another explanation for CV0’s superior performance in Orchard A, particularly for FCC and CMR, is that the assumption of perfect covariance among trees from the same family does not apply because Orchard A is a seedling seed orchard. Members of the same family are either half-siblings or full-siblings but never clones. This is different from Orchards B and C, where the assumption of perfect covariance among trees from the same family is valid.

These findings suggest that multi-source models may suffer from overfitting when trained on observations from the same environment they intend to predict. Therefore, in some cases it may be more effective to ignore family-specific information (as M1 model does) and even avoid relying on phenotypic observations of those families in that environment (as in the CV0 scenario). Another cause of overfitting is the complexity of the models: A reduced model incorporating only fall multispectral imagery and the genetic background (M6) matched or exceeded the full model M7 across all traits and cross-validation scenarios. This parsimony result is consistent with the overlapping detected during the variance components analysis (**Figure 3**, **Supplementary Table 3**). The superior generalization of M6 suggests that the additional interaction terms in M7 introduce noise rather than signal under sparse data conditions, and that a parsimonious fall multispectral model provides more stable estimates across environments. The use of regularization techniques could help complex models to reduce overfitting in environments with lower sample sizes. From an operational standpoint, fall imagery, unlike foliar sampling, requires only one non-destructive flight per orchard. Therefore, its use would be justified if the modest gain over the baseline model with no predictor information (~2–6%) is economically viable.

### Predictor datasets as environmental proxies - the CV0 case

One key distinction between CV2 and CV0 is that CV2 allows models to be trained on observations of the target environment. This enables the estimation of environment distribution parameters. The environment mean (or intercept) is critical for RMSE because a systematic bias in predictions inflates this metric. This is particularly important when the goal is to approximate actual phenotypic values, rather than rankings.

When prompted to predict an unseen environment (CV0 scenario), all models regressed toward the training population mean, making environments with extreme averages harder to predict (**Figure 6**). However, models incorporating microenvironmental covariates deviated from this pattern more effectively and notably differed from those that were better at the accuracy-measured tasks. For instance, M6 ranked genetic backgrounds most accurately (**Figures 4A**, **5A**), but did not recover environmental intercepts as well as other models (M0 for ICC, M5 for FCC and M2 for CMR), as shown in **Figure 6**, **Table 3**.

### Modeling limitations and further research

This study highlights the importance of genetic background information for accurate predictions. However, the use of such information was limited because trees were nested within orchards, meaning genetic information could only be shared within an orchard across years, not between orchards. Additionally, CV0 (prediction of unseen environments) standard errors were systematically higher than those of CV2 and CV1 and further elevated for 2020 environments where ~80% of observations were missing; those results should be validated as more data become available.

A second limitation concerns Orchard A, where half-sib families are modeled as clones (covariance = 1), whereas true covariances are δ = 0.5 for full-sibs and δ = 0.25 for half-sibs; a proper genomic relationship matrix would be required to correctly partition genetic variance in this seedling orchard (Resende et al., 2012; Munoz et al., 2014; Li et al., 2018). Genomic data could also enable a better understanding of the genotype-by-predictor interactions, but expectations should be moderated, since genomic selection in conifers has generated considerable methodological interest but limited evidence of routine operational deployment, particularly for reproductive traits across poorly connected environments.

## Conclusions

This study explored the potential of tree-level multi-source datasets as predictors of complex conelet-related traits in three lodgepole pine orchards under various predictive scenarios. These findings showed that interactions between multi-source data and genetic backgrounds explained a greater proportion of phenotypic variability than multi-source datasets alone, and that the primary driver of predictive accuracy was the presence of identical genetic backgrounds in both training and testing sets. Multi-source datasets alone showed limited potential for predicting the performance of unobserved genetic backgrounds and cannot substitute for genomic or pedigree information under a CV1-like scenario.

Among the predictor datasets examined, fall multispectral imagery showed the most promise for capturing genotype-specific responses across environments. A model incorporating fall multispectral imagery and its interaction with the genetic background term (E+L+EL+F+FL) matched or exceeded the full model across all traits and cross-validation scenarios. The fall multispectral interaction term consistently explained more phenotypic variance than its spring counterpart, suggesting that fall canopy status captures more of the genotype-specific environmental response. This advantage was specific for some trait-scenario combinations: fall multispectral data contributed meaningfully to initial and final conelet count prediction in both CV2 and CV0 but provided no benefit for conelet mortality rate in the CV0 scenario, where the best strategy remained averaging across other environments with genotype-by-environment corrections.

## Data Availability

All experimental data (phenotypic, phenomic, and environmental) has been fully deposited and made publicly available on Zenodo: https://zenodo.org/records/22112958. The data is also provided within the **Supplementary Material**.

## Supplementary material

Supplementary Table 1 Location of HOBO data loggers used in the CRD project and the number of sensors used each year in the three lodgepole pine seed orchards. Sensors were removed from Orchard A in fall 2020 and remained in the other two orchards in association with the supplemental breeding experiment when mature cones were collected in Fall 2021.

Supplementary Table 2 Vegetation index, meaning of terms, the equation used to calculate the vegetation indices (Gallardo-Salazar and Pompa-García, 2020), and purpose of the index.

Supplementary Table 3 Variance components and relative contributions to phenotypic variation. The first column represents the trait, ICC (Initial Conelet Count), FCC (Final Conelet Count), and CMR (Conelet Mortality Rate), respectively. The terms used in the models are E (environment, year-location combination), N (nutrients), S (multispectral spring), F (multispectral fall), M (microenvironment), L (genetic background). The two-letter terms represent interactions (e.g., NL is nutrients x genotype interaction). M7: full model is E+L+EL+M+N+S+F+ML+NL+SL+FL.
