# Supplementary material for: Prediction of conelet mortality rates in interior lodgepole pine using phenomic, nutritional, and environmental data
Source: Front Plant Sci. 2026 Oct 8;17:1845181. doi: 10.3389/fpls.2026.1845181 (PMC13687373; doi:10.3389/fpls.2026.1845181)
Supplement: Supplementary file 1 [file SupplementaryFile1.docx]

**SUPPLEMENTARY MATERIAL**

**Supplementary Table 1.** Location of HOBO data loggers used in the CRD project and the number of sensors used each year in the three lodgepole pine seed orchards. Sensors were removed from Orchard A in fall 2020 and remained in the other two orchards in association with the supplemental breeding experiment when mature cones were collected in Fall 2021.

| **Orchard** | **Row** | **Position** | **Sensor**  **#** | **Sensors**  **2019** | **Sensors**  **2020** | **Sensors**  **2021** |
| --- | --- | --- | --- | --- | --- | --- |
| A | 7 | 92 | 1 |  | X |  |
|  | 10 | 123 | 2 |  | X |  |
|  | 8 | 255 | 3 |  | X |  |
|  | 23 | 49 | 4 | X | X |  |
|  | 22 | 119 | 5 | X | X |  |
|  | 24 | 226 | 6 |  | X |  |
|  | 41 | 19 | 7 |  | X |  |
|  | 41 | 162 | 8 |  | X |  |
|  | 39 | 244 | 9 |  | X |  |
|  | 56 | 19 | 10 | X | X |  |
|  | 56 | 171 | 11 | X | X |  |
|  | 55 | 219 | 12 |  | X |  |
| B | 4 | 7 | 25 | X | X | X |
|  | 4 | 27 | 26 | X | X | X |
|  | 4 | 49 | 27 |  | X | X |
|  | 11 | 7 | 28 |  | X | X |
|  | 11 | 24 | 29 |  | X | X |
|  | 12 | 41 | 30 |  | X | X |
|  | 19 | 11 | 31 |  | X | X |
|  | 20 | 25 | 32 |  | X | X |
|  | 19 | 39 | 33 |  | X | X |
|  | 27 | 9 | 34 | X | X | X |
|  | 28 | 23 | 35 |  | X | X |
|  | 28 | 38 | 36 | X | X | X |
| C | 5 | 10 | 13 | X | X | X |
|  | 7 | 29 | 14 |  | X | X |
|  | 7 | 46 | 15 | X | X | X |
|  | 18 | 11 | 16 |  | X | X |
|  | 18 | 28 | 17 |  | X | X |
|  | 19 | 46 | 18 |  | X | X |
|  | 31 | 12 | 19 |  | X | X |
|  | 32 | 38 | 20 |  | X | X |
|  | 32 | 49 | 21 |  | X | X |
|  | 45 | 15 | 22 | X | X | X |
|  | 44 | 30 | 23 |  | X | X |
|  | 46 | 46 | 24 |  | X | X |

**Supplementary Table 2**. Vegetation index, meaning of terms, the equation used to calculate the vegetation indices (Gallardo-Salazar and Pompa-García 2020), and purpose of the index.

| **Vegetation**  **index** | **Meaning** | **Equation** | **Purpose** |
| --- | --- | --- | --- |
| NDVI | Normalized Difference Vegetation Index | 𝑁𝐼𝑅 − 𝑅𝐸𝐷  𝑁𝐼𝑅 + 𝑅𝐸𝐷 | It indicates **vegetation growth and nutrients** (chlorophyll content in vegetation). |
| GNDVI | Green Normalized Difference  Vegetation Index | 𝑁𝐼𝑅 − 𝐺𝑅𝐸𝐸𝑁  𝑁𝐼𝑅 + 𝐺𝑅𝐸𝐸𝑁 | It shows whether **vegetation is lacking water or nutrients** or is suffering from post-maturity **biomass recession**. |
| LCI | Leaf Chlorophyll Index | 𝑁𝐼𝑅 − 𝑅𝑒𝑑𝐸𝑑𝑔𝑒  𝑁𝐼𝑅 + 𝑅𝐸𝐷 | It evaluates vegetation growth and yield, and assesses **plant nutrient stress**, disease, and aging. |
| NDRE | Normalized Difference Red- edge Index | 𝑁𝐼𝑅 − 𝑅𝑒𝑑𝐸𝑑𝑔𝑒  𝑁𝐼𝑅 + 𝑅𝑒𝑑𝐸𝑑𝑔𝑒 | It can be used for managing vegetation based on variables such as chlorophyll and sugar content. |
| OSAVI | Optimized Soil Adjusted Vegetation  Index | 𝑁𝐼𝑅 − 𝑅𝐸𝐷  𝑁𝐼𝑅 + 𝑅𝐸𝐷 + 0.16 | It is used for removing the impact of soil conditions on vegetation. |
| RVI | Ratio Vegetation Index | 𝑁𝐼𝑅  𝑅𝐸𝐷 | It indicates the presence of vegetation and correlates with the amount of vegetation in particular fields. Its values can increase far beyond 1; very high values are generally on the order of 30. |
| TVI | Transformed Vegetation Index | 𝑁𝐼𝑅 − 𝑅𝐸𝐷  + 0.5  𝑁𝐼𝑅 + 𝑅𝐸𝐷 | Same meaning as NDVI, but the 0.5 constant helps avoid operating with negative values. |
| NDGI | Normalized Difference Greenness Index | 𝐺𝑅𝐸𝐸𝑁 − 𝑅𝐸𝐷  𝐺𝑅𝐸𝐸𝑁 + 𝑅𝐸𝐷 | It provides information about **differences** in the content of **various pigments** and variation throughout the entire vegetation season. |

| **Trait** | **Model** | **E** | | **L** | | | **EL** | **M** | | **ML** | **N** | **NL** | **S** | | **SL** | **F** | **FL** | **residual** |
| --- | --- | --- | --- | --- | --- | --- | --- | --- | --- | --- | --- | --- | --- | --- | --- | --- | --- | --- |
| **ICC** | M0 | 28.77 (14.8%) | | 24.29 (12.5%) | | | 7.50 (3.9%) |  | |  |  |  |  | |  |  |  | 134.25 (68.9%) |
|  | M1 | 33.24 (16.7%) | | |  | |  | 0.24 (0.1%) | |  | 0.42 (0.2%) |  | 0.89 (0.4%) | |  | 0.85 (0.4%) |  | 163.72 (82.1%) |
|  | M2 | 29.23 (15.0%) | | 25.23 (12.9%) | | | 4.89 (2.5%) | 0.18 (0.1%) | |  | 0.28 (0.1%) |  | 0.60 (0.3%) | |  | 0.65 (0.3%) |  | 133.91 (68.7%) |
|  | M3 | 28.73 (14.6%) | | 24.52 (12.5%) | | | 5.79 (3.0%) | 0.24 (0.1%) | | 2.42 (1.2%) |  |  |  | |  |  |  | 134.64 (68.6%) |
|  | M4 | 29.38 (14.8%) | | 24.26 (12.2%) | | | 5.48 (2.8%) |  | |  | 0.36 (0.2%) | 8.53 (4.3%) |  | |  |  |  | 130.38 (65.7%) |
|  | M5 | 26.62 (13.6%) | | 23.56 (12.0%) | | | 3.88 (2.0%) |  | |  |  |  | 0.94 (0.5%) | | 11.85 (6.0%) |  |  | 129.30 (65.9%) |
|  | M6 | 30.52 (15.3%) | | 19.59 (9.8%) | | | 3.61 (1.8%) |  | |  |  |  |  | |  | 0.67 (0.3%) | 20.16 (10.1%) | 124.56 (62.6%) |
|  | M7 | 29.27 (14.5%) | | 19.17 (9.5%) | | | 2.08 (1.0%) | 0.11 (0.1%) | | 1.41 (0.7%) | 0.18 (0.1%) | 5.32 (2.6%) | 0.34 (0.2%) | | 6.57 (3.3%) | 0.31 (0.2%) | 14.68 (7.3%) | 121.68 (60.5%) |
| **FCC** | M0 | 29.21 (19.8%) | | 17.49 (11.8%) | | | 5.13 (3.5%) |  | |  |  |  |  | |  |  |  | 95.81 (64.9%) |
|  | M1 | 32.57 (21.5%) |  | | | |  | 0.19 (0.1%) | |  | 0.31 (0.2%) |  | 0.55 (0.4%) | |  | 0.55 (0.4%) |  | 117.15 (77.4%) |
|  | M2 | 29.58 (20.0%) | | 18.41 (12.4%) | | | 3.27 (2.2%) | 0.14 (0.1%) | |  | 0.22 (0.2%) |  | 0.48 (0.3%) | |  | 0.46 (0.3%) |  | 95.30 (64.5%) |
|  | M3 | 28.75 (19.4%) | | 17.67 (11.9%) | | | 3.70 (2.5%) | 0.19 (0.1%) | | 1.96 (1.3%) |  |  |  | |  |  |  | 95.91 (64.7%) |
|  | M4 | 30.26 (20.1%) | | 17.36 (11.5%) | | | 3.77 (2.5%) |  | |  | 0.28 (0.2%) | 6.27 (4.2%) |  | |  |  |  | 92.78 (61.6%) |
|  | M5 | 27.45 (18.4%) | | 17.80 (12.0%) | | | 2.88 (1.9%) |  | |  |  |  | 0.64 (0.4%) | | 8.21 (5.5%) |  |  | 91.97 (61.7%) |
|  | M6 | 31.31 (20.6%) | | 14.68 (9.7%) | | | 2.73 (1.8%) |  | |  |  |  |  | |  | 0.50 (0.3%) | 13.12 (8.6%) | 89.46 (58.9%) |
|  | M7 | 29.85 (19.5%) | | 14.92 (9.8%) | | | 1.54 (1.0%) | 0.09 (0.1%) | | 1.14 (0.7%) | 0.14 (0.1%) | 3.95 (2.6%) | 0.25 (0.2%) | | 4.88 (3.2%) | 0.23 (0.2%) | 9.11 (6.0%) | 86.77 (56.8%) |
| **CMR** | M0 | 194.89 (26.9%) | | 76.66 (10.6%) | | | 28.72 (4.0%) | |  |  |  |  |  | |  |  |  | 424.80 (58.6%) |
|  | M1 | 207.86 (27.7%) | | | |  |  | 3.01 (0.4%) | |  | 1.29 (0.2%) |  | 1.80 (0.2%) | |  | 2.20 (0.3%) |  | 533.21 (71.2%) |
|  | M2 | 183.11 (25.4%) | | 79.19 (11.0%) | | | 20.48 (2.8%) | 6.09 (0.8%) | |  | 0.92 (0.1%) |  | 1.41 (0.2%) | |  | 1.30 (0.2%) |  | 427.77 (59.4%) |
|  | M3 | 185.64 (25.9%) | | 74.60 (10.4%) | | | 14.32 (2.0%) | 3.85 (0.5%) | | 19.79 (2.8%) |  |  |  | |  |  |  | 419.69 (58.5%) |
|  | M4 | 195.05 (26.6%) | | 76.20 (10.4%) | | | 22.05 (3.0%) | |  |  | 1.19 (0.2%) | 36.55 (5.0%) | |  |  |  |  | 403.50 (54.9%) |
|  | M5 | 189.74(25.7%) | | 77.70 (10.5%) | | | 22.39 (3.0%) | |  |  |  |  | 1.79 (0.2%) | | 33.60 (4.6%) |  |  | 412.51 (55.9%) |
|  | M6 | 197.68 (26.7%) | | 63.40 (8.6%) | | | 23.23 (3.1%) | |  |  |  |  |  | |  | 1.78 (0.2%) | 54.54 (7.4%) | 399.41 (54.0%) |
|  | M7 | 187.71 (25.3%) | | 62.97 (8.5%) | | | 9.29 (1.3%) | 4.29 (0.6%) | | 14.22 (1.9%) | 0.60 (0.1%) | 25.00 (3.4%) | 0.91 (0.1%) | | 21.45 (2.9%) | 0.88 (0.1%) | 39.57 (5.3%) | 376.25 (50.6%) |

**Supplementary Table 3.** Variance components and relative contributions to phenotypic variation. The first column represents the trait, ICC (Initial Conelet Count), FCC (Final Conelet Count), and CMR (Conelet Mortality Rate), respectively. The terms used in the models are E (environment, year-location combination), N (nutrients), S (multispectral spring), F (multispectral fall), M (microenvironment), L (genetic background). The two-letter terms represent interactions (e.g., NL is nutrients x genotype interaction). M7: full model is E+L+EL+M+N+S+F+ML+NL+SL+FL
